# Humour processing in frontotemporal lobar degeneration: A behavioural and neuroanatomical analysis

**DOI:** 10.1016/j.cortex.2015.03.024

**Published:** 2015-08

**Authors:** Camilla N. Clark, Jennifer M. Nicholas, Susie M.D. Henley, Laura E. Downey, Ione O. Woollacott, Hannah L. Golden, Phillip D. Fletcher, Catherine J. Mummery, Jonathan M. Schott, Jonathan D. Rohrer, Sebastian J. Crutch, Jason D. Warren

**Affiliations:** a Dementia Research Centre, UCL Institute of Neurology, University College London, London, United Kingdom; b London School of Hygiene and Tropical Medicine, University of London, London, United Kingdom

**Keywords:** Humor, Cartoons, Frontotemporal lobar degeneration, Frontotemporal dementia, Semantic dementia, Incongruity

## Abstract

Humour is a complex cognitive and emotional construct that is vulnerable in neurodegenerative diseases, notably the frontotemporal lobar degenerations. However, humour processing in these diseases has been little studied. Here we assessed humour processing in patients with behavioural variant frontotemporal dementia (*n* = 22, mean age 67 years, four female) and semantic dementia (*n* = 11, mean age 67 years, five female) relative to healthy individuals (*n* = 21, mean age 66 years, 11 female), using a joint cognitive and neuroanatomical approach. We created a novel neuropsychological test requiring a decision about the humorous intent of nonverbal cartoons, in which we manipulated orthogonally humour content and familiarity of depicted scenarios. Structural neuroanatomical correlates of humour detection were assessed using voxel-based morphometry. Assessing performance in a signal detection framework and after adjusting for standard measures of cognitive function, both patient groups showed impaired accuracy of humour detection in familiar and novel scenarios relative to healthy older controls (*p* < .001). Patient groups showed similar overall performance profiles; however the behavioural variant frontotemporal dementia group alone showed a significant advantage for detection of humour in familiar relative to novel scenarios (*p* = .045), suggesting that the behavioural variant syndrome may lead to particular difficulty decoding novel situations for humour, while semantic dementia produces a more general deficit of humour detection that extends to stock comedic situations. Humour detection accuracy was associated with grey matter volume in a distributed network including temporo-parietal junctional and anterior superior temporal cortices, with predominantly left-sided correlates of processing humour in familiar scenarios and right-sided correlates of processing novel humour. The findings quantify deficits of core cognitive operations underpinning humour processing in frontotemporal lobar degenerations and suggest a candidate brain substrate in cortical hub regions processing incongruity and semantic associations. Humour is a promising candidate tool with which to assess complex social signal processing in neurodegenerative disease.

## 1 Introduction

Humour is among the most ubiquitous and highly valued of social phenomena, and the sense of humour is at once a complex cognitive construct and a basic source of empathy and cohesion with our fellows (McGhee, 1979; Vrticka, Black, & Reiss, 2013). Whereas the neurology of laughter has been studied in some detail (Provine, 2001; Rohrer, Warren, & Rossor, 2009), the neural architecture of humour appreciation and the impact of brain disease on that architecture are less well understood. Humour often entails the juxtaposition of apparently incompatible or ambiguous elements that cohere in a surprising way to link psychological expectancies with pleasure and reward (Chan, Chou, Chen, & Liang, 2012; Chan, Chou, Chen, Yeh, et al., 2012; Suls, 1972). Accordingly, the brain mechanisms that process humour are also likely *a priori* to be involved in analysing other kinds of complex social signals. Indeed, functional neuroimaging and electrophysiological studies in the healthy brain have implicated distributed fronto-temporo-parietal cortical and subcortical dopaminergic mesolimbic networks in processing cognitive aspects of humour and associated emotions of surprise and delight (Goel & Dolan, 2001; Mobbs, Greicius, Abdel-Azim, Menon, & Reiss, 2003; Moran, Wig, Adams, Janata, & Kelley, 2004; Vrticka et al., 2013; Wild et al., 2006). Diverse developmental and acquired brain disorders involving this circuitry produce deficits of humour perception, comprehension or emotional response (Bihrle, Brownell, Powelson, & Gardner, 1986; Braun, Lussier, Baribeau, & Ethier, 1989; Corcoran, Cahill, & Frith, 1997; Eddy, Mitchell, Beck, Cavanna, & Rickards, 2011; Gardner, Ling, Flamm, & Silverman, 1975; Girardi, Macpherson, & Abrahams, 2011; Samson & Hegenloh, 2010; Shammi & Stuss, 1999; Staios et al., 2013).

Neurodegenerative diseases on the frontotemporal lobar degeneration (FTLD) spectrum commonly affect social cognition and might therefore be anticipated to produce disturbances of humour processing. The behavioural variant of frontotemporal dementia (bvFTD) is a paradigmatic acquired disorder of social cognition (Rascovsky et al., 2011) while semantic dementia (SD) and other syndromes associated with anterior temporal lobe atrophy erode knowledge of social concepts alongside other kinds of conceptual knowledge (Zahn, Moll, Iyengar, et al., 2009). Various abnormalities of humour processing and behaviours, including impaired sarcasm detection and compulsive punning or ‘Witzelsucht’, have been described in these patients (Ehrlé, Henry, Pesa, & Bakchine, 2011; Ibanez & Manes, 2012). Clinical experience suggests that altered sense of humour (particularly a predilection for the more fatuous comedic forms of farce, pranks and scatological jokes) commonly accompanies bvFTD, while humourlessness may develop in association with syndromes of predominant temporal lobe atrophy (Chan et al., 2009). Such disturbances of humour may be early features of disease (Warren, Rohrer, & Rossor, 2013), but remain poorly understood and difficult to characterise. Aside from its relevance to clinical symptoms, humour is an attractive candidate model with which to analyse the neuropsychological and neurobiological bases of social cognitive dysfunction in these syndromes. Difficulty shifting perspective and impaired use of context may underpin inter-personal difficulties of various kinds experienced by patients with FTLD (Ibanez & Manes, 2012). Humour (which often relies on perspective shifts) is likely *a priori* to be a sensitive index of these processes. Cartoon stimuli have been used to probe theory of mind processing and sarcasm in patients with bvFTD and SD (Ehrlé et al., 2011; Irish, Hodges, & Piguet, 2014; Lough et al., 2006; Snowden et al., 2003). However, such processes are themselves complex constructs and potentially vulnerable to associated cognitive deficits (such as verbal semantic impairment), besides any more specific impairment of humour processing *per se*. While there are a number of theories and cognitive models of humour comprehension (Chan, Chou, Chen, & Liang, 2012; Chan, Chou, Chen, Yeh, et al., 2012; McGhee, 1979; Morreall, 2013; Suls, 1972; Vrticka et al., 2013), most include a requirement for logical resolution of apparently incongruous elements. Detection and resolution of incongruity is a generic function of fronto-temporo-parietal networks implicated in bvFTD and SD (Chan, Chou, Chen, Yeh, et al., 2012; Michelon, Snyder, Buckner, McAvoy, & Zacks, 2003; Watanabe et al., 2014; Zhou, Gennatas, Kramer, Miller, & Seeley, 2012). Accurate analysis of incongruity, ambiguity and conflict may be particularly critical for decoding social signals, potentially accounting for the fundamental role of humour processing during human social development (Gervais & Wilson, 2005; Neely, Walter, Black, & Reiss, 2012), as well as deficits of mentalising and higher-order social cognition exhibited by patients with bvFTD when required to determine humorous intent (Kipps, Nestor, Acosta-Cabronero, Arnold, & Hodges, 2009).

Here we investigated humour processing and its neuroanatomical correlates in a cohort of patients with bvFTD and SD as well as healthy older individuals. Our objective was to assess generic cognitive operations that are engaged by humour: namely, detection and resolution of conflicting sensory and conceptual information, particularly as embodied in social contexts (Eslinger et al., 2007; Krueger et al., 2009); processing of familiar objects and concepts from semantic memory (Zahn, Moll, Iyengar, et al., 2009), cognitive flexibility and the processing of novelty (Irish, Piguet, & Hodges, 2011; Kramer et al., 2007). We created a novel neuropsychological test requiring a decision about the humorous intent of nonverbal cartoons, in which we manipulated orthogonally the humour content and familiarity of the depicted scenarios. Our experimental design was intended to deconstruct key ‘building blocks’ of humour cognition that might be engaged by more complex operations (such as theory of mind), rather than indexing those operations directly. Our design was motivated by cognitive models of humour processing (Degabriele & Walsh, 2010; Vrticka et al., 2013) that emphasise resolution of incongruity as a unifying principle of humour comprehension. In addition, by manipulating the familiarity of humorous scenarios, we sought to separate cognitive processes underpinning the resolution of incongruities based on prior associations (stock comedic situations) from processes that mediate active reinterpretation of incongruities to achieve a surprising resolution (novel scenarios). Familiar humorous scenarios might be regarded as a component of social conceptual knowledge for which a tentative brain organisation has been defined centred on the nondominant anterior temporal lobe (Zahn, Moll, Iyengar, et al., 2009; Zahn, Moll, Paiva, et al., 2009). In practice, the distinction between familiar and novel humorous scenarios maps broadly onto a distinction between scenarios that represent incongruous physical elements (a key characteristic of more primitive, childlike or ‘slapstick’ humour) versus scenarios that juxtapose incongruous psychological elements such as concepts, beliefs or motivations (a characteristic of more mature humour) (Vrticka et al., 2013). In line with our emphasis on humour cognition, we did not in this study address the behavioural or brain correlates of amusement *per se*. While obviously integral to humour in daily life, the cognitive analysis of humour is dissociable from the emotional response to humorous stimuli and likely to be separately vulnerable to the effects of neurodegenerative disease, on both clinical and neuroanatomical grounds (Bartolo, Benuzzi, Nocetti, Baraldi, & Nichelli, 2006; Downey et al., 2013; Mensen, Poryazova, Schwartz, & Khatami, 2014). Based on their core syndromic characteristics and previous evidence relating to humour processing in these syndromes (Ibanez & Manes, 2012; Irish et al., 2014; Lough et al., 2006; Snowden et al., 2003; Warren et al., 2013; Zahn, Moll, Iyengar, et al., 2009), we hypothesised that syndromes of bvFTD and SD are associated with impaired humour comprehension with differentiable neuropsychological deficits linked to the processing of novelty and familiarity respectively, in humorous scenarios. More specifically, we predicted that patients with bvFTD would have particular difficulty processing less familiar scenarios, involving active decoding of the elements of the cartoon, but might show relatively preserved ability to process highly familiar scenarios such as those based on childlike or ‘slapstick’ humour. Whereas patients with SD might show a more general loss of humour comprehension extending across both familiar and novel scenarios.

Neuroanatomical correlates of humour cognition were assessed using voxel-based morphometry (VBM). Drawing on previous neuroimaging evidence to guide a region-of-interest analysis (Goel & Dolan, 2001; Mobbs et al., 2003; Moran et al., 2004; Neely et al., 2012; Wild et al., 2006), we hypothesised regional grey matter correlations of altered humour processing in a distributed brain network including temporo-parieto-occipital junction, anterior temporal lobe, ventromedial prefrontal and anterior cingulate cortex. Within this network, certain key ‘hubs’ have been identified. Cortical areas in the region of the temporo-parieto-occipital junction (especially in the left cerebral hemisphere) may mediate humour detection and analysis of potentially humorous (in particular, incongruous) stimuli, based on prior expectations and stored concepts (Coulson & Kutas, 2001; Franklin & Adams, 2011; Goel & Dolan, 2001; Gold & Buckner, 2002; Moran et al., 2004; Neely et al., 2012; Schurz, Aichhorn, Martin, & Perner, 2013; Shammi & Stuss, 1999; Thompson-Schill, D'Esposito, Aguirre, & Farah, 1997; Wild et al., 2006). Accordingly, we hypothesised that detection of incongruity in our cartoon stimuli would be particularly associated with grey matter volume in this region. Antero-medial and ventral temporal lobe areas and their inferior frontal lobe projections are likely to be engaged in humour comprehension, resolution of incongruity and semantic (including social conceptual) evaluation (Bartolo et al., 2006; Chan, Chou, Chen, Yeh, et al., 2012; Mobbs et al., 2003; Moran et al., 2004; Samson, Hempelmann, Huber, & Zysset, 2009; Samson, Zysset, & Huber, 2008; Zahn, Moll, Iyengar, et al., 2009; Zahn, Moll, Paiva, et al., 2009). We therefore hypothesised a grey matter correlate of humour category processing (familiar versus novel cartoon scenarios) in this region. Ventromedial prefrontal cortex and anterior cingulate have been implicated in linking salient (especially, apparently incompatible or surprising) sensory and cognitive features of humorous stimuli with emotional coding of ‘funniness’ and more specifically in the analysis of mental states embodied in humour (Coulson & Kutas, 2001; Du et al., 2013; Kohn, Kellermann, Gur, Schneider, & Habel, 2011). Therefore, we hypothesised a grey matter correlate in this region for the processing of novel cartoon scenarios that might entail a psychological perspective shift.

## 2 Methods

### 2.1 Participant groups

Thirty-three patients fulfilling current consensus criteria for bvFTD (Rascovsky et al., 2011); (*n* = 22, mean age 67 years, standard deviation (SD) 7.7 years, four female) or SD [the semantic variant of primary progressive aphasia: (Gorno-Tempini et al., 2011) (*n* = 11, mean age 67 years, SD 7.7 years, five female)] were recruited via a tertiary specialist cognitive clinic; 21 healthy older individuals (mean age 66 years, SD 5 years, 11 female) with no history of neurological or psychiatric illness also participated. Participant characteristics are summarised in Table 1. All participants had lived most of their adult lives and the majority had also grown up (to age 16 years) in the United Kingdom. Participants had a comprehensive general neuropsychological assessment including standard measures of visual perceptual, executive, semantic and social cognition functions. These included the object decision subtest of the Visual Object and Spatial Perception (VOSP) battery; the Trails test (used to assess task-switching); the British Picture Vocabulary Scale (BPVS, a general cross-modal measure of semantic memory) and the size-weight attributes test [a within-modality measure of visual object knowledge (Warrington & Crutch, 2007)], see Supplementary Material on-line; and the Awareness of Social Inference Test [TASIT, requiring decoding of sarcastic intent (McDonald et al., 2006)]. Neuropsychological findings corroborated the clinical syndromic diagnosis in all cases. The patient cohort included 12 cases with confirmed pathogenic mutations (five MAPT, seven C9orf72). Cerebrospinal fluid analysis or 18F-amyloid (Florbetapir) PET imaging (performed as part of another study) in 10 other cohort members provided no evidence for underlying Alzheimer's disease.

Ethical approval for the study was obtained from the local institutional ethics committee and written informed consent was obtained from all participants in accordance with the Declaration of Helsinki.

### 2.2 Experimental design

To assess humour processing we designed a series of simple non-verbal cartoons, each requiring a forced-choice decision (whether or not the scenario was intended to be humorous). This design reflected our primary focus on the cognitive elements of humour. We did not, for example, ask participants in the experiment proper to rate their subjective amusement for each cartoon, as emotional and cognitive components of humour processing are likely to dissociate, particularly in patients with the target diseases. Four conditions were combined in a factorial design comprising cartoons that were intended to be either humorous or non-humorous and to represent familiar or novel scenarios. The experimental design allowed us to control stimulus characteristics between cartoon conditions while minimising any dependence on language processing.

**Fig. 1:**
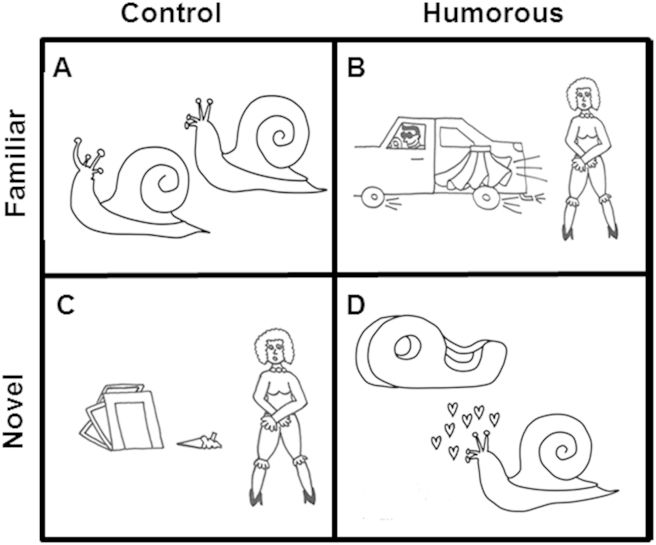
Examples of captionless cartoon stimuli representing each experimental condition. Non-humorous control (A, C) and humorous (B, D) cartoon categories were designed to share structural elements. Surface features of humorous cartoons were rearranged to create familiar congruous (A) or unresolvably incongruous (C) non-humorous control scenarios. Control familiar scenarios contained congruous elements, while familiar humorous scenarios contained incongruities that could be labelled as humorous based on prior cultural associations (compare panels A and B). Control novel scenarios contained incongruities that were not resolvable, while humorous scenarios contained surface incongruities that were ultimately resolvable in a surprising and amusing way (compare panels C and D). Within the category of humorous cartoons, familiar humorous scenarios had more prominent stock elements of farce and slapstick with incongruities based on characters' physical actions or attributes; while novel humorous scenarios had incongruities based on incompatible concepts or beliefs, and resolution required an active perspective shift by the viewer (compare panels B and D).

Following review of published cartoon collections directed at adults or children, scenarios employing non-verbal humour were adapted or generated de novo by a single artist (CNC) to create an initial set of 180 new cartoons. All were line drawings without captions, each comprising a single frame depicting human and/or animal characters interacting with each other or with the physical environment. ‘Familiar’ humorous cartoons were designed to depict stock comedic situations, variants of which appear frequently in Western culture (e.g., the central character suffers some misadventure, such as slipping on a banana peel or having undergarments exposed in public); while ‘novel’ humorous cartoons were designed to depict novel comedic scenarios relying on some active shift in viewer perspective (e.g., a snail declares his love for a tape dispenser; see examples in Fig. 1). Familiar humorous cartoons emphasised conventionally incongruous physical (‘slapstick’) elements; resolvable as humorous, based on previously learned associations; whereas novel humorous cartoons emphasised resolution of apparently incongruous concepts as humorous, based on interpretation of characters' beliefs or motives. Structural elements of humorous cartoons were rearranged to create matching non-humorous control cartoons balanced for perceptual features, semantic associations of individual elements and affective cues such as facial expressions. Control cartoons for familiar humorous scenarios depicted commonly-encountered, congruous everyday scenarios not normally considered humorous, while control cartoons for novel humorous scenarios depicted bizarre incongruities that lack any clear resolution (see Fig. 1).

Based on data in a pilot group of 14 healthy older individuals (details in Supplementary Material on-line), a subset of cartoons was selected such that each achieved >75% consensus on whether it represented a humorous or non-humorous scenario. The final experimental stimulus set of 60 cartoons comprised: i) familiar humorous scenarios, containing incongruous elements that were resolvable based on prior association (*n* = 10); ii) novel humorous scenarios, containing elements that were superficially incongruous but resolvable (*n* = 10); iii) familiar control (non-humorous) scenarios, containing fully congruous elements (*n* = 20); iv) novel control (non-humorous) scenarios, containing incongruous elements that were not clearly resolvable (*n* = 20). This classification was supported by pilot control ratings (summarised in Table S1 in Supplementary Material on-line). Cartoons representing humorous scenarios were rated by pilot controls as significantly (*p* < .001) more amusing than control cartoons representing non-humorous scenarios, while familiarity of the cartoon scenarios differed significantly between conditions (*p* < .001, in ascending order of familiarity: novel control < novel humorous < familiar control < familiar humorous). In addition, cartoons depicting familiar humorous scenarios were rated as having significantly more prominent elements of physical humour (generally associated with farce or ‘slapstick’) than cartoons depicting novel humorous scenarios (*p* < .001).

### 2.3 Experimental procedures

Stimuli were presented on the monitor screen of a notebook computer running Matlab7^®^. Trials were delivered in fully randomised order that varied for each individual. The task on each on trial was to decide whether or not the cartoon was intended to show ‘a joke’ with a single ‘Yes/No’ response. Prior to commencing the experiment, practice examples (not used subsequently in the experiment), representing each condition, were shown to familiarise participants with the stimuli and it was established that each participant understood the task. During the experiment no feedback about performance was given and no time limits were imposed. Participant responses were recorded for offline analysis.

### 2.4 Behavioural data analysis

All behavioural data analyses were conducted using Stata12^®^. Demographic characteristics and neuropsychological and behavioural rating data were compared between participant groups using Fisher's exact test for categorical variables, and for continuous variables either two sample *t*-tests or Wilcoxon rank-sum tests where assumptions for the *t*-test were materially violated (for example, due to skewed data distribution).

A mixed effects logistic regression model incorporating all participants' binary responses was used to model scores on the experimental humour decision task for each experimental group. Bias is often a significant issue in patients with executive or frontal lobe impairment, especially if (as here) response probabilities are not balanced across conditions (i.e., ‘hits’ are relatively infrequent). Accordingly, to take account of any bias introduced by patient factors or the imbalance of trial numbers between humorous and control conditions, a framework based on signal detection theory was used to fit a logistic regression model for odds of labelling a cartoon as humorous (de Carlo, 1998). The dependent variable was a binary category indicating for each cartoon whether or not each participant in a group had labelled the cartoon as humorous. Participant-level random effects were included to account for the repeated-measures nature of the data and the model included a random intercept and random coefficient for cartoon type (humorous versus non-humorous). Accordingly, this model assessed humour detection accuracy as odds ratios comparing labelling of humorous and non-humorous cartoons across all participants in each group. Here, an odds ratio of 1 corresponds to chance level performance, i.e., the group had equal likelihood of labelling a humorous or control cartoon as humorous; an odds ratio >1 corresponds to increased accuracy discriminating humorous from control cartoons; and an odds ratio <1 corresponds to over-rejection of humorous cartoons as non-humorous or over-labelling of control cartoons as humorous. An interaction of humour with familiarity across cartoon conditions was fitted to allow calculation of odds ratios of humour detection within familiar scenarios (between familiar humorous and familiar control cartoon conditions); within novel scenarios (between novel humorous and novel control conditions); and between humour conditions.

In order to take account of potentially confounding, extraneous effects on processing of these cartoon stimuli, the regression model incorporated covariates of age, gender, years of education, Trails making task (B−A difference score; as an executive and disease severity index) and the object decision subtest of the VOSP battery (as an index of visual perceptual ability). We separately assessed associations of humour detection score with visual semantic memory functions, as indexed by BPVS and (within the SD group alone) size/weight attribution test scores; with social cognition function as indexed by TASIT score; with general intellectual performance, as indexed by Mini-Mental State Examination (MMSE) score; and with estimated symptom duration. A threshold *p* < .05 was accepted as the criterion for statistical significance in all analyses.

### 2.5 Brain image acquisition and VBM analysis

Brain MRI data were acquired for 28 patients (19 bvFTD, nine SD) on a Siemens Trio 3 T MRI scanner using a 32-channel phased array head-coil and a sagittal 3-D magnetization prepared rapid gradient echo T1 weighted volumetric sequence (echo time/repetition time/inversion time = 2.9/2200/900 ms, dimensions 256 × 256 × 208, voxel size 1.1 × 1.1 × 1.1 mm). Volumetric brain images were assessed visually in all planes to ensure adequate coverage and to exclude artefacts or significant motion. Pre-processing of patient brain MR images was performed using the Segment routine and the DARTEL toolbox of SPM12 (Asburner, 2007; Ridgway et al., 2008, fil.ion.ucl.ac.uk/spm/). Normalisation, segmentation and modulation of grey and white matter images used default parameter settings, with a smoothing Gaussian kernel of full-width-at-half-maximum 6 mm. Smoothed segments were warped into MNI space using the “Normalise to MNI” routine. In order to adjust for individual differences in global grey matter volume during subsequent analysis, total intracranial volume (TIV) was calculated for each participant by summing grey matter, white matter and cerebrospinal fluid volumes following segmentation of all three tissue classes. A study-specific mean brain image template, for displaying results, was created by warping all bias-corrected native space whole-brain images to the final DARTEL template in MNI space and calculating the average of the warped brain. To help protect against voxel drop-out due to marked local regional atrophy, a customised explicit brain mask was made based on a specified ‘consensus’ voxel threshold intensity criterion (Ridgway et al., 2009), whereby a particular voxel was included in the analysis if grey matter intensity at that voxel was >.1 in >70% of participants (rather than in all participants, as with the default SPM mask). The mask was applied to the smoothed grey matter segments prior to statistical analysis.

Using the framework of the general linear model, multiple regression was used to examine associations between grey matter volume and humour variables of interest. In separate design matrices, voxel intensity (an index of grey matter volume) was modelled as a function of log-transformed odds ratios indexing overall accuracy of humour detection, accuracy of detection of humour in familiar scenarios, and accuracy of detection of humour in novel scenarios. In all models, age, gender, TIV, patient group and Trails B−A performance were included as nuisance covariates. For each model, separate contrasts (one-tailed *t*-tests) assessed linear associations between grey matter and humour score of interest across the combined patient cohort and within the larger bvFTD group alone; both positive and negative (inverse) associations were assessed.

Statistical parametric maps were thresholded at two levels of significance: *p* < .05 after family-wise error (FWE) correction for multiple voxel-wise comparisons over the whole brain; and *p* < .05 after FWE correction for multiple comparisons within anatomical regions of interest based on our prior anatomical hypotheses. Anatomical small volumes were derived from the Oxford–Harvard brain maps (Desikan et al., 2006) in FSLview (Jenkinson, Beckmann, Behrens, Woolrich, & Smith 2012) and boundaries edited using MRIcron (mccauslandcenter.sc.edu/mricro/mricron/) to conform to the study template (participant mean) brain image. These small volumes included key areas implicated in humour processing in the healthy brain for the contrasts of interest. Our small volume analysis was based on the prior assumption that neuroanatomical substrates for key cognitive operations underpinning humour processing are at least potentially dissociable. Accordingly, contrasts on humour detection performance were separately assessed within small volumes comprising lateral temporo-occipital-parietal junctional cortex [previously implicated in detection of incongruity in potentially humorous stimuli: (Neely et al., 2012; Wild et al., 2006)], temporal lobe anterior to Heschl's gyrus [previously implicated in semantic evaluation of humorous stimuli: (Mobbs et al., 2003; Samson et al., 2008; Wild et al., 2006)] and ventromedial prefrontal cortex [previously implicated in processing behavioural and inter-personal relevance of humour: (Goel & Dolan, 2007; Samson et al., 2009, 2008)]. Anatomical regions are displayed in Fig. S1 in Supplementary Material on-line.

## 3 Results

### 3.1 General characteristics of participant groups

Participant groups were matched for age, gender and socio-cultural background and patient groups did not differ significantly in clinical disease duration. Patients had, on average, significantly fewer years of education than healthy control participants and this factor was incorporated as a covariate in subsequent analyses (Table 1). However, absolute differences in educational attainment were small and all participant groups were relatively highly educated.

### 3.2 Behavioural data: humour decision task

**Fig. 2:**
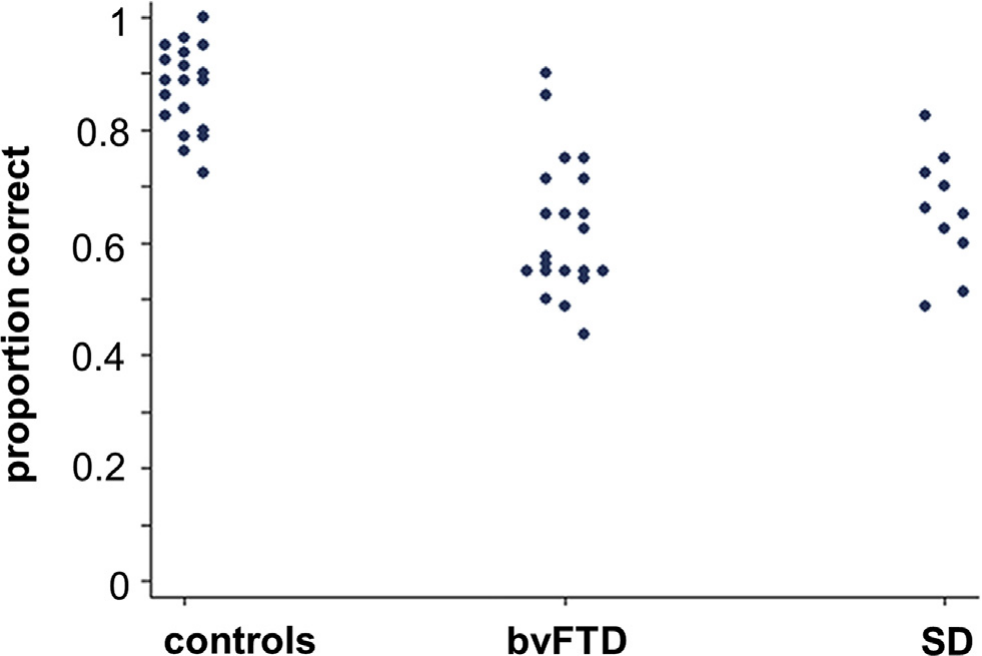
Individual raw scores on the humour decision task. Proportion trials correct is shown for each participant (based on overall score/60); proportion correct .5 corresponds to chance performance. bvFTD, patients with behavioural variant frontotemporal dementia; controls, healthy controls; SD, patients with semantic dementia.

Performance data on the humour decision task for each participant group are summarised in Table 2 Individual raw scores are plotted in Fig. 2 and further details are provided in Table S2 in Supplementary Material on-line.

### 3.3 Humour detection

On overall humour detection (discrimination of humorous from non-humorous cartoons), both the bvFTD group (odds ratio 4.9, 95% confidence interval 2.1–11) and the SD group (odds ratio 5.7, 95% confidence interval 2.4–13) performed above chance, but significantly worse (*p* < .001) than the healthy control group. There was no significant performance difference between patient groups. However, comparing raw performance data in each condition between the patient groups (Table S2) revealed that patients with bvFTD tended to over-label novel control cartoons as humorous, whereas patients with SD tended to reject familiar humorous cartoons as non-humorous. Assessed in relation to general demographic and cognitive factors, humour detection accuracy over the combined participant cohort was not associated with age (*p* = .45), gender (*p* = .71), years of education (*p* = .37) or visuoperceptual function (VOSP score; *p* = .28), but showed a significant positive association with executive function (Trails B−A score; *p* = .03). Neither the healthy control group nor the combined patient cohort showed a significant correlation between humour detection accuracy and BPVS score (controls *p* = .31, patients *p* = .24); while the SD group additionally showed no correlation between humour detection accuracy and nonverbal semantic (size-weight attributes test score; *p* = .14). There was no significant correlation between humour detection accuracy and TASIT score (*p* = .68). Humour detection accuracy was not correlated with symptom duration (*p* = .85) but was correlated with MMSE score (*p* = .01) over the patient cohort.

On humour detection within familiar scenarios (discrimination of familiar humorous scenarios from familiar non-humorous scenarios), both the bvFTD group and the SD group performed above chance (odds ratios for humour detection, 7.3 and 5.6 respectively), but were significantly worse (*p* < .001) than the healthy control group. On humour detection within novel (incongruous) scenarios (discrimination of novel humorous scenarios from novel non-humorous scenarios), a similar pattern was again observed for both bvFTD and SD groups (odds ratios for humour detection, 3.5 and 5.5 respectively; *p* < .001 versus healthy control performance). There were no significant performance differences between the patient groups.

### 3.4 Humour category differentiation

For the comparison between humour categories, the healthy control group and SD group showed no significant performance discrepancy for humour detection in familiar versus novel scenarios; whereas the bvFTD group showed a significant advantage for detection of humour in familiar relative to novel scenarios (odds ratio 1.57, 95% confidence interval 1.01–2.45, *p* = .045) and a trend toward a performance difference compared with the healthy control group (*p* = .058). There was no significant performance difference between the patient groups.

### 3.5 Neuroanatomical data

**Fig. 3:**
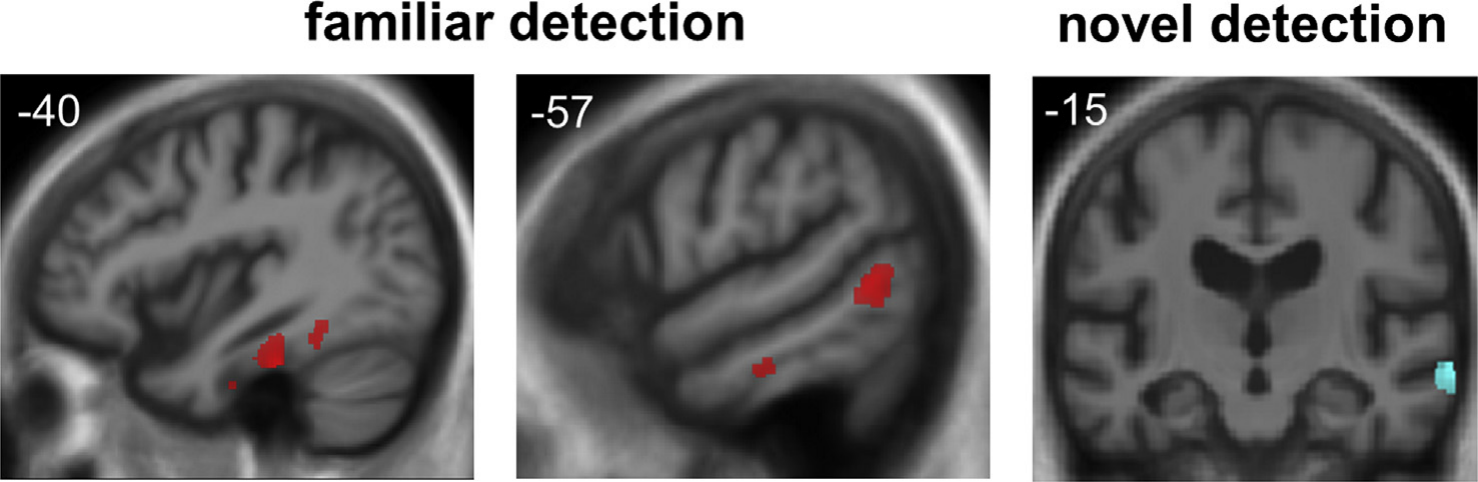
Statistical parametric maps (SPMs) of regional grey matter volume associated with humour processing (shown here for the behavioural variant frontotemporal dementia group; see also Table 3). Correlates of processing familiar humour (relying on recognition of stock comedy situations, exemplified by farce) are coded in red and correlates of processing novel humour (relying on a psychological perspective shift, exemplified by satire) are coded in cyan. **familiar detection**, grey matter volume positively correlated with accuracy of detecting humour in familiar scenarios from humour decision task (see Table 2); **novel detection**, grey matter volume positively correlated with accuracy of detecting humour in novel scenarios from humour decision task. Results are overlaid on sections of the normalised study-specific T1-weighted mean brain MR image. The MNI coordinate (mm) of the plane of the section is indicated and the coronal section shows the right hemisphere on the right. SPMs are thresholded at *p* < .05 after family-wise error correction for multiple comparisons within small volumes of interest (see online material, Supplementary Fig. 1).

Associations between grey matter volume and humour processing in the patient cohort are summarised in Table 3. Statistical parametric maps are presented in Fig. 3 and data plots of correlations of peak voxel parameter values with humour indices are presented in Fig. S2 in Supplementary Material on-line. All contrasts are reported at a statistical significance threshold *p* < .05 (after FWE correction for multiple comparisons within pre-specified anatomical small volumes of interest).

No significant associations between grey matter volume and experimental contrasts of interest were identified at threshold *p* < .05_FWE_ after correction for multiple comparisons over the whole brain. Examined at threshold *p* < .05 after correction for multiple comparisons within the pre-specified anatomical regions of interest, no significant associations were identified between grey matter volume and overall humour detection accuracy. However, humour detection accuracy within familiar scenarios was positively correlated with grey matter volume in the left fusiform gyrus in the combined patient cohort and additionally with grey matter volume in lateral temporo-occipital junctional cortex within the bvFTD group. Humour detection accuracy within novel scenarios was positively correlated with grey matter volume in right anterior middle temporal gyrus and superior temporal sulcus within the bvFTD group. No other significant grey matter associations were identified.

## 4 Discussion

Here we have demonstrated deficits of humour comprehension in two canonical syndromes of FTLD, bvFTD and SD. Both syndromes showed impaired detection of humorous intent in both familiar and novel scenarios, corresponding broadly to farcical or ‘slapstick’ versus ‘psychological’ humour, respectively. Patients with bvFTD showed a clear advantage for comprehension of familiar (farcical) compared with novel (psychological) humorous scenarios. This contrasted with the equivalent performance of healthy older individuals and patients with SD across humour categories. There were additional, qualitative differences comparing the performance profiles of the two patient groups. Patients with bvFTD had greater difficulty distinguishing novel ‘bizarre’ scenarios from humorous ones, whereas patients with SD had greater difficulty detecting humour in stock comedic situations. Taken together, these profiles suggest that bvFTD is particularly associated with impaired detection of humour where this relies on the active deconstruction of a novel incongruous situation, while SD is associated with a more general defect of humour detection that extends to familiar scenarios that we normally ‘learn’ as humorous during social development (Degabriele & Walsh, 2010; Neely et al., 2012). These findings extend previous work suggesting abnormalities of humour processing in FTLD (D. Chan et al., 2009; Ehrlé et al., 2011; Ibanez & Manes, 2012; Irish et al., 2014; Warren et al., 2013). Our experimental design, based on manipulation of relatively simple, nonverbal cartoons, allowed us to assess key elements in humour comprehension (novelty and incongruity) relatively independently of potentially confounding verbal, semantic and executive performance factors.

A neuroanatomical analysis identified distributed cortical signatures of altered humour comprehension in the present patient cohort. Taken together, these structural associations corroborate previous functional neuroanatomical evidence in the healthy brain for separable neural mechanisms of particular cognitive components of humour processing (Du et al., 2013; Franklin & Adams, 2011; Goel & Dolan, 2001; Mobbs et al., 2003; Moran et al., 2004; Vrticka et al., 2013; Wild et al., 2006). Detection of humour in familiar scenarios was associated with relative preservation of grey matter in a left-sided cortical network including fusiform gyrus and lateral temporo-occipital cortex. This network is likely to represent fundamental attributes of humorous (or potentially humorous) stimuli, particularly if (as in the relevant contrast here) humour detection rests on detection of incongruity. Fusiform gyrus has previously been implicated as an interface in the analysis of cartoons and other complex visual stimuli for resolution of potentially conflicting elements, coherent cross-modal linkage with stored semantic concepts (for example, stock comedic situations) and associated emotional resonance (Goel & Dolan, 2001; Watanabe et al., 2014). Indeed, stimulation of left fusiform may generate a sense of mirth (Arroyo et al., 1993). A closely related set of functions may be subserved by lateral temporo-occipital junctional cortex (Bartolo et al., 2006; Goel & Dolan, 2001; Mobbs et al., 2003; Samson et al., 2008). This region is activated by stimuli perceived as funny by young children (Neely et al., 2012) and in initial decoding of incongruities used by adults in perceiving slapstick humour (Wild et al., 2006), in line with the present paradigm in which familiar humorous scenarios contained prominent elements of physical (slapstick) comedy (Neely et al., 2012). Detection of humour in novel scenarios here was associated (in the bvFTD group) with relative preservation of grey matter in right-sided, antero-lateral superior temporal cortex. This correlate of novelty processing in humour might be regarded as a nondominant hemisphere analogue of the more posteriorly extending network in the dominant hemisphere, associated with processing familiar humorous scenarios. Anterior right superior and middle temporal cortex may engage social conceptual knowledge in a process of conflict resolution (Zahn, Moll, Iyengar, et al., 2009; Zahn, Moll, Paiva, et al., 2009), perhaps more specifically accessing learned associations or stored conceptual knowledge about potentially comedic situations (Bartolo et al., 2006; Goel & Dolan, 2001; Mobbs et al., 2003; Samson et al., 2008).

The brain mechanisms of altered humour processing suggested by these behavioural and neuroanatomical data may be of wider relevance to the phenomenology of FTLD syndromes. With respect to detection of humour, the requirement to process incongruity may index impaired ability to detect and resolve ambiguity and conflict in the world at large. These are likely to be generic features of both bvFTD and SD and may occur early in the course of disease (Barense et al., 2010; Eslinger et al., 2007; Krueger et al., 2009; McMillan et al., 2013), mapping onto deficits of social understanding in the face of ambiguous or conflicting information (Chiong et al., 2013; Kipps et al., 2009). Our findings in FTLD suggest an analogy with previous reports in patients undergoing temporal lobectomy who were no longer able to detect humour in cartoons due to impaired integration of situational elements and deficient perspective shifting (Ferguson, Schwartz, & Rayport, 1969). Impaired ability to resolve incongruity might reflect generic deficits in maintaining and monitoring alternative possible resolutions or in integrating the elements of a scene to achieve coherence; or a more specific deficit in engaging social semantic templates (Barense et al., 2010; Zahn, Moll, Iyengar, et al., 2009). Temporal lobe networks implicated in FTLD are likely to play a key role in all of these processes (Goel & Dolan, 2001). With respect to the processing of novel humorous scenarios, cognitive flexibility and the capacity to shift perspective or cognitive set are also likely to be key vulnerabilities in FTLD (Kramer et al., 2007; McMillan, Rascovsky, Khella, Clark, & Grossman, 2012; Perri, Monaco, Fadda, Caltagirone, & Carlesimo, 2014). This is exemplified by reduced empathy in social situations (Le Bouc et al., 2012; Rankin et al., 2006), which is a defining feature of bvFTD (Rascovsky et al., 2011).

Theory of mind has been emphasised in previous accounts of humour processing (Franklin & Adams, 2011) and indeed, cartoons have been used to index theory of mind processes in FTLD (Irish et al., 2014; Lough et al., 2006; Snowden et al., 2003). While cartoons here (particularly within the novel humour set) incorporated elements of theory of mind, our paradigm did not manipulate mentalising factors primarily or require an explicit decision about characters' mental states. Rather, our emphasis here was on generic cognitive operations that might link humour to other neuropsychological and behavioural deficits. Moreover, theory of mind is difficult to assess reliably in patients (like those with SD) who have severe verbal deficits. It is noteworthy that a structural neuroanatomical analysis here revealed a relative dearth of classical theory of mind correlates in prefrontal cortices. While a more overtly social context might engage such regions (Franklin & Adams, 2011), the profile of prefrontal cortical activation revealed by previous functional neuroimaging studies of humour is variable (Samson et al., 2009). It may be that more posterior and ventral temporal and parietal junctional cortices and their projections constitute a critical network for humour processing. With particular reference to FTLD, deficits of complex cognitive processes such as moral reasoning have been shown to arise from aberrant interaction of large-scale brain networks (Chiong et al., 2013), suggesting a candidate mechanism via which more posterior cortical zones (not generally regarded as core targets of FTLD) may play a critical role in humour processing in these syndromes. The present findings predict alterations of humour processing in other neurodegenerative disorders such as Alzheimer's disease that disrupt interactions between large-scale brain networks (Le Bouc et al., 2012): these alterations may differ qualitatively from those in FTLD [for example, over-identification with characters' plights: (Sturm et al., 2013)]. It should also be emphasised that the cerebral correlates of theory of mind continue to be defined and these are likely to overlap extensively with temporal lobe regions involved in semantic and affective analysis (Irish et al., 2014), including the anterior superior temporal cortical region identified here as a correlate of novelty processing in humour.

From a clinical perspective, the present findings provide a basis for understanding the altered humour behaviours exhibited by patients with FTLD and align these neurodegenerative disorders with diseases causing focal brain damage in which abnormalities of humour processing and behaviour (including humourlessness and context-inappropriate humour) have been previously described (Bihrle et al., 1986; Ferguson et al., 1969; Gardner et al., 1975; Shammi & Stuss, 1999). Our findings resonate with the complaints of caregivers of patients with FTLD, frequently indicating that they have lost the ability to appreciate more subtle comedy, that their tastes in comedy have coarsened or that they have become humourless or more inclined to find humour in inappropriate contexts. Altered humour sensibility may constitute a distinct domain of social cognitive function that is not well captured by standard neuropsychological instruments, and impaired humour processing may contribute importantly to behavioural difficulties more generally, including the flouting of social norms (Barsuglia et al., 2014). While bvFTD and SD were both associated with extensive abnormalities of humour processing, our findings suggest that relatively greater affinity for more fatuous or childlike humour may be a marker of bvFTD while SD produces a more general impairment of humour processing, opening up avenues for assessing these syndromes that could be further explored at the bedside.

This study has several limitations that should help guide future work. Our deliberately reductionist approach should be extended to other genres of humour [for example, wordplay, absurdist: (Samson et al., 2009)] and other comedic contexts including more naturalistic social settings (Franklin & Adams, 2011). Humour is complex and multidimensional; a thorough understanding of this phenomenon will entail the study of the cognitive operations that allow us to explain why something is funny and the subjective experience of amusement. Anatomical region-of-interest analyses are potentially susceptible to bias. Larger patient cohorts (recruited for example by collaborating specialist centres) should be studied to improve power to detect effects at the level of the whole brain and to compare bvFTD, SD and other canonical clinical syndromes (in particular, Alzheimer's disease). To define brain mechanisms of aberrant humour processing and emotional responses more fully will require correlation of cognitive and behavioural measures with functional neuroanatomical techniques, including electrophysiological methods such as magnetoencephalography that can capture temporal dynamics. These should be guided by specific hypotheses: for example, the present study [in line with previous evidence: (Goel & Dolan, 2001)] predicts the existence of a ‘lexicon’ of humorous scenarios that might have a cognitive organisation analogous to other semantic domains (Omar, Hailstone, Warren, Crutch, & Warren, 2010). Affective components of humour were not examined in our study, but are likely to be critical for the normal integration of humour behaviours in daily life. This emotional dimension of humour should be addressed alongside cognitive processes in future studies. Incorporation of techniques to measure autonomic responses may be a useful means of objectifying affective valuation in humour, particularly in patients with dementia who may have difficulty in reporting emotional states. Longitudinal analyses are particularly called for to assess the biomarker potential of humour indices in detecting and tracking disease: this is an area of early promise based on the present behavioural data and observations in presymptomatic genetic cohorts (Dopper et al., 2014), but more detailed stratification in larger cohorts will be required to account for wide individual variation in processing humour (see Fig. 2) and to assess the clinical value of metrics of humour processing. Ultimately, correlation with histopathological and molecular data will be required. With these caveats in mind, we propose humour as a novel, clinically and neurobiologically relevant model of complex social signal processing in neurodegenerative disease. The unique cultural and cognitive status humour enjoys might be exploited to probe complex behavioural deficits that would otherwise remain inaccessible.

## Figures and Tables

**Figure:**
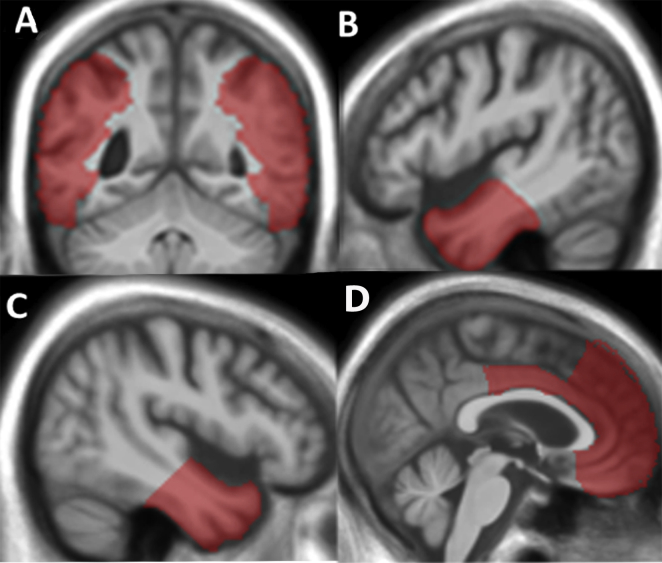


**Figure:**
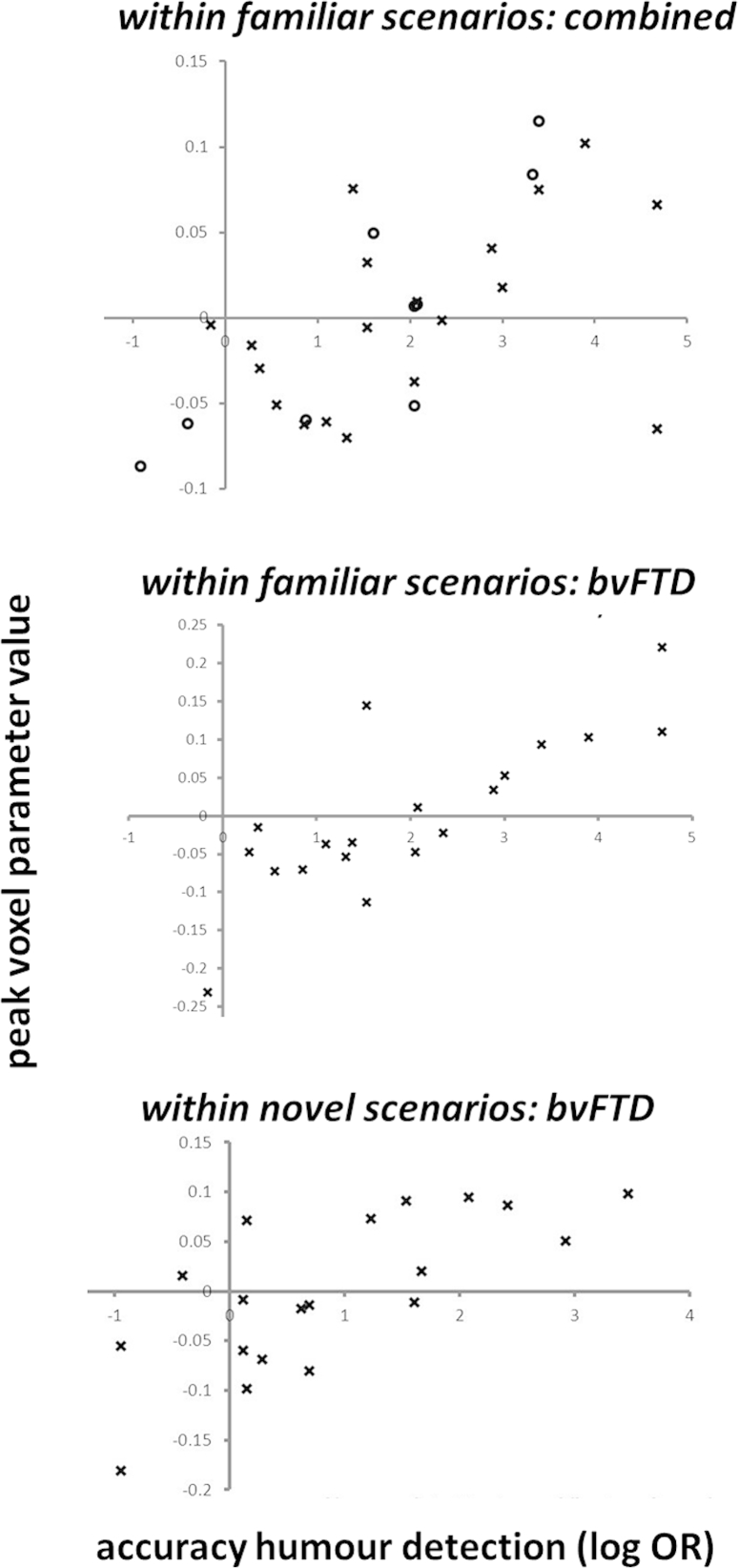


**Table 1:** Summary of participant demographic, clinical and general neuropsychological characteristics.

Characteristic	Healthy controls	bvFTD	SD
**General**			
No., gender (M:F)	21 (10:11)	22 (18:4)	11 (6:5)
Handedness (L:R)	N/A	01:20	01:10
Age (yrs)	66 (5)	67 (7.7)	67 (7.7)
Education (yrs)	15.7 (1.9)	**13.9 (3.0)**	**13.1 (2.5)**
Background (UK&Eire:other)^a^	19:2^b^	19:3^c^	10:1^d^
Symptom duration (yrs)	N/A	9 (5.4)	5.5 (3.0)
**General intellect**			
MMSE (/30)	N/A	25 (3.5)	18 (8.1)
VIQ	123 (6.4)	84 (20.6)*	69 (15.5)*
PIQ	126 (9.7)	**98 (19.6)**	**107 (20.2)**
WASI vocabulary (/80)^e^	71.4 (3.8)	38.5 (20.1)*	23.1 (19.8)*
WASI block design (/71)	33.4 (18.7)	40 (19.3)	38.2 (18.5)
WASI similarities (/38)	42.1 (3.3)	25.2 (11.1)*	15.5 (10.6)*
WASI matrices (/42)	26.8 (2.9)	**17.1 (7.5)**	21.7 (6.9)
**Language and literacy functions**			
GNT (/30)	27.8 (1.9)	10.5 (9.3)*	1.1 (2.2)*
Reading (NART) (/50)	44.1 (3.0)	**29.2 (12.9)**	**22.4 (19.0)**
Arithmetic (GDA) (/24)	15.1 (4.4)	**10 (7.6)**	**9.3 (7.5)**
**Short term and episodic memory**			
Digit span forward (/12)	8.9 (2.0)	7.9 (2.2)	6.6 (2.4)
Digit span reverse (/12)	7.3 (1.9)	6.4 (2.2)	6.3 (3.0)
RMT words (/50)	47.6 (2.2)	**34.3 (7.3)**	**31.3 (7.2)**
RMT faces (/50)	45.8 (5.0)	**32.8 (7.0)**	**34.3 (11.2)**
**Semantic memory**			
BPVS (/150)	147.9 (1.8)	129.7 (17.7)*	78.3 (46.3)*
Size-weight attributes^f^(/60)	57.4 (2.3)	N/A	49.1 (11.6)^g^
**Executive functions**			
Verbal fluency (/min)	16.3 (4.7)	**8 .3 (3.9)**	**7.4 (5.3)**
Stroop (ink colour) (sec)	53.7 (10.8)	**98.9 (41.2)**	**96.8 (54.1)**
Trails (B−A difference) (sec)	36 (24)	**140 (89)**	**113 (98)**
**Visual perceptual functions**			
VOSP object decision (/20)	18.5 (1.7)	17.2 (1.8)	**16.9 (2.4)**
Unusual views (20)	17 (2.3)	**10 (4.5)**	**7 (6.1)**
Usual views (20)	20 (.3)	**17 (3.8)**	**17 (2.5)**
**Social cognition**			
TASIT (Emotion) (/14)	11.4 (.7)	**6.8 (2.5)**	N/A
TASIT (Social inference) (/36)	31.4 (2.2)	**20.9 (5.4)**	N/A

Mean (standard deviation) scores are shown unless otherwise indicated; maximum scores are shown after tests (in parentheses). Bold denotes mean difference between patient and control group statistically significant (*p* < .05). *Mean difference between patient groups statistically significant (*p* < .05). bvFTD, behavioural variant frontotemporal dementia; GNT, Graded Naming Test (McKenna & Warrington, 1983); GDA, Graded Difficulty Arithmetic (Jackson M & Warrington, 1986); MMSE, Mini-Mental State Examination score; N/A, not assessed; NART, National Adult Reading Test (Nelson, 1982); PIQ, performance IQ; RMT, Recognition Memory Test (E. K. Warrington, 1984); SD, semantic dementia; Stroop D-KEFS, Delis Kaplan Executive System (Delis, Kaplan, & Kramer, 2001); Trails-making task (B−A difference score) based on maximum time achievable 2.5 min on task A, 5 min on task B (Lezak, Howieson, & Loring, 2004); average of fluency tasks with letters F,A,S each within 1 min (Gladsjo et al., 1999); VIQ, verbal IQ; VOSP, Visual Object and Spatial Perception Battery (E.K. Warrington & James, 1991); WASI, Wechsler Abbreviated Scale of Intelligence (Wechsler, 1997); Size-weight attributes test (E. K. Warrington & Crutch, 2007), further details in Supplementary Material on-line.

a Where lived to age 16.

b One North America, one other Western European country.

c One North America, two other Western European countries.

d One South Africa; BPVS, British Picture Vocabulary Scale (Dunn LM, Whetton, & Pintilie, 1982).

e Total score referenced to age range 56–83 years.

f Scores referenced to separate historical healthy control group (*n* = 40; age range 45–79 years; Professor EK Warrington, personal communication).

g 7 patients completed this test.

**Table 2:** Summary of humour decision task performance for all participant groups.

Condition comparison		Healthy controls	bvFTD	SD
Humour detection: overall^a^	OR	90**	4.9**	5.7**
	CI	41–193	2.1–11	2.4–13
Humour detection: familiar scenarios^b^	OR	93**	7.3**	5.6**
	CI	32–267	2.6–20	2.0–15
Humour detection: novel scenarios^c^	OR	104**	3.5**	5.5**
	CI	38–285	1.4–8.9	2.1–15
Humour category^d^	OR	0.8	1.6*	1.0
	CI	.5–1.4	1.01–2.5	.6–1.7

Odds ratios (ORs) with 95% confidence intervals (CI) are shown for key condition comparisons of interest in the behavioural analysis of group performance on the humour decision task. Comparisons index participant performance on aspects of humour processing (see text for further details); bold denotes patient performance significantly different from healthy controls. *Significantly different from chance (*p* < .05). **Significantly different from chance (*p* < .01).

a OR > 1 indicates increased accuracy in labelling any humorous cartoon as humorous compared with control cartoons.

b OR > 1 indicates increased accuracy in labelling familiar humorous scenarios as humorous compared with control scenarios matched for familiarity.

c OR > 1 indicates increased accuracy in labelling novel humorous scenarios as humorous compared with control scenarios matched for familiarity.

d OR > 1 indicates greater accuracy in labelling familiar compared with novel humorous cartoons.

**Table 3:** Summary of neuroanatomical associations of humour processing in the patient cohort.

Contrast	Region	Side	Cluster (voxels)	Peak coordinates (mm)			Z score	*P* value	Group
				*x*	*y*	*z*			
Humour detection: familiar scenarios^a^	Fusiform gyrus	L	59	−40	−30	−29	3.87	.048	Combined
	Fusiform gyrus	L	597	−40	−30	−29	4.25	.013	bvFTD
	T – O junction	L	419	−57	−54	1	4.06	.041	
Humour detection: novel scenarios^b^	Ant MTG/STS	R	315	68	−15	−12	4.47	.008	bvFTD

All statistically significant associations between grey matter volume and humour parameters are summarised (see also Fig. 3). Local maxima coordinates are in MNI standard stereotactic space. All contrasts were significant at threshold *p* < .05 after family-wise error correction within the pre-specified anatomical small volume of interest. ‘Combined’ refers to common associations across both patient groups. Contrasts index patient performance on aspects of humour processing, as follows (see also Table 2).

a Grey matter volume positively correlated with humour detection accuracy in familiar scenarios.

b Grey matter volume positively correlated with humour detection accuracy in novel scenarios. Ant, anterior; bvFTD, behavioural variant of frontotemporal dementia; MTG, middle temporal gyrus; STS, superior temporal sulcus; T-O, temporo-occipital.
