# Supplementary material for: Humour processing in frontotemporal lobar degeneration: A behavioural and neuroanatomical analysis
Source: Cortex. 2015 Aug;69:47–59. doi: 10.1016/j.cortex.2015.03.024 (PMC4534772; doi:10.1016/j.cortex.2015.03.024)
Supplement: Supplementary file 1 [file mmc1.docx]

**SUPPLEMENTARY MATERIAL**

**Humour processing in frontotemporal lobar degeneration: a behavioural and neuroanatomical analysis,** by CN Clark et al

**Pilot experiment**

Fourteen healthy older individuals (mean age 60 (s.d. 4.1) years, eight female) with no history of neurological or psychiatric illness and of British or Western European cultural background participated in the initial pilot experiment. For each cartoon stimulus (n=180), participants were asked to decide whether or not it was intended to be humorous, whether or not the scenario depicted was familiar and (on a Likert scale), how amusing and how heavily the humour relied on actions. Participant responses were recorded for offline analysis. Based on these data a subset of 60 cartoons was selected for presentation in the main experiment; characteristics of the experimental stimulus set are summarised in Table S1. Data were analysed using Wilcoxon rank sum test as data were non-normally distributed.

Subsequent *post hoc* testing established strong consensus between this pilot control group and the main experimental healthy control group in classifying cartoon scenarios as humorous versus non-humorous (inter-group correlation Spearman’s correlation coefficient = 0.70, p <0.001) and categorisation of joke stimuli as familiar versus unfamiliar (Spearman’s correlation coefficient = 0.89, p < 0.001).

**Size-weight attributes test** ([E. K. Warrington & Crutch, 2007](#_ENREF_77))

This test probed attribute knowledge of animals and objects within the visual domain, a within-modality index of semantic memory (as a control for semantic processing of cartoon stimuli in patients with semantic dementia). Participants were presented with 30 picture triads depicting familiar animals or inanimate objects; for animal stimuli, the task on each trial was to decide which member of the triad was largest and which smallest, while for object stimuli the task was to decide which was heaviest and which lightest.

**Table S1.** Summary of characteristics of the experimental stimulus conditions

| **Cartoon condition** | **No.** | **Category^a^** | **Familiarity^b^** | **Amusement^c^** | **Physicality^c^** |
| --- | --- | --- | --- | --- | --- |
| **Humour familiar** | 10 | 1.94 (0.06) | 1.86 (0.12) | 2.69 (0.21) | 3.96 (0.29) |
| **Humour novel** | 10 | 1.92 (0.06) | 1.52 (0.11) | 3.06 (0.44) | 3.29 (0.49) |
| **Control familiar / congruous** | 20 | 1.06 (0.06) | 1.68 (0.21) | 1.14 (0.12) | N/A |
| **Control novel / incongruous** | 20 | 1.08 (0.06) | 1.35 (0.19) | 1.14 (0.11) | N/A |

Behavioural rating data were obtained from the pilot group of 14 healthy older individuals; mean (standard deviation) values are shown. Category ratings (1, not intended to be humorous; 2, intended to be humorous) and familiarity ratings (1, unfamiliar scenario; 2, familiar scenario) were based on a binary classification; amusement ratings (1 = not at all amusing, 5 = very amusing) and physicality ratings (1 = humour not at all reliant on physical actions, 5 = humour very reliant on physical actions) were based on 5-point Likert scales. **a**, humour and control condition ratings significantly different (p<0.001) **b**, ratings for each condition significantly different (p<0.001) from all other conditions; **c**, familiar and novel humour condition ratings significantly different (p<0.001); N/A, not applicable.

**Table S2.** Humour decision task performance data by condition for participant groups

| **Condition** | **Healthy controls** | **bvFTD** | **SD** |
| --- | --- | --- | --- |
| **Familiar humorous** | 0.78 (0.17) | 0.65 (0.32) | 0.57 (0.22) |
| **Novel humorous** | 0.81 (0.15) | 0.56 (0.28) | 0.57 (0.17) |
| **Familiar control** | 0.96 (0.05) | 0.71 (0.24) | 0.73 (0.21) |
| **Novel control** | 0.95 (0.07) | 0.59 (0.31) | 0.73 (0.18) |

Scores are shown as proportions (where 0.5 is chance performance) by group, with standard deviations in parentheses. See text for further details of conditions and Figure 2 for individual data plots. bvFTD, behavioural variant frontotemporal dementia; SD, semantic dementia

**Figure S1.** Representative sections of anatomical regions used for multiple voxel-wise comparisons correction in region-of-interest analyses based on prior anatomical hypotheses (see Introduction and Methods). Regions of interest are rendered on sections of the average normalised brain template for the entire patient cohort undergoing VBM; the right hemisphere is shown on the right in coronal sections. Panel **A,** right and left lateral temporo-occipital-parietal junctional cortex; **B,** left temporal lobe anterior to Heschl’s gyrus; **C,** right temporal lobe anterior to Heschl’s gyrus; **D,** ventromedial prefrontal and orbitofrontal cortex and anterior cingulate.

**Figure S2.** Data plots derived from the VBM analysis showing correlations of peak voxel parameter estimate values (effect sizes) with log-transformed odds ratios (OR) for humour detection in familiar cartoon scenarios by the combined patient cohort (top panel) and within the behavioural variant frontotemporal dementia (bvFTD) group alone middle panel) and humour detection in novel cartoon scenarios by the bvFTD group alone. Corresponding peak voxel coordinates are listed in Table 3. Crosses signify individual patients with bvFTD; open circles (top panel) signify individual patients with semantic dementia.
